# Evolution and maintenance of microbe‐mediated protection under occasional pathogen infection

**DOI:** 10.1002/ece3.6555

**Published:** 2020-07-16

**Authors:** Anke Kloock, Michael B. Bonsall, Kayla C. King

**Affiliations:** ^1^ Department of Zoology University of Oxford Oxford UK

**Keywords:** defensive symbiosis, experimental evolution, heterogeneity, host–pathogen interactions, protection

## Abstract

Every host is colonized by a variety of microbes, some of which can protect their hosts from pathogen infection. However, pathogen presence naturally varies over time in nature, such as in the case of seasonal epidemics. We experimentally coevolved populations of *Caenorhabditis elegans* worm hosts with bacteria possessing protective traits (*Enterococcus faecalis*), in treatments varying the infection frequency with pathogenic *Staphylococcus aureus* every host generation, alternating host generations, every fifth host generation, or never. We additionally investigated the effect of initial pathogen presence at the formation of the defensive symbiosis. Our results show that enhanced microbe‐mediated protection evolved during host‐protective microbe coevolution when faced with rare infections by a pathogen. Initial pathogen presence had no effect on the evolutionary outcome of microbe‐mediated protection. We also found that protection was only effective at preventing mortality during the time of pathogen infection. Overall, our results suggest that resident microbes can be a form of transgenerational immunity against rare pathogen infection.

## Graphical abstract

Microbe‐mediated protection is evolved even under rare pathogen infection. This evolution is independent of initial pathogen presence and the interval of pathogen infection.

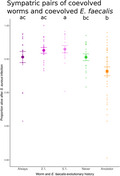

## 1 INTRODUCTION

In nature, all plants and animals are colonized by microbes (Barrière, & Feĺix, 2005; Ley, Peterson, & Gordon, 2006; Vántus, Kovács, & Zsolnai, 2014). The composition of these microbial communities is highly diverse and includes harmful, neutral, and beneficial microbial species (Ley et al., 2006), including those that can be important players in host defense against parasites, a phenomenon referred to as “defensive mutualism” (King, 2019; May & Nelson, 2014). Recognized for over a century, defensive mutualism has been observed in plants (Mendes et al., 2011) and in a range of animals (Dillon, Vennard, & Charnley, 2000; Dong, Manfredini, & Dimopoulos, 2009; Jaenike, Unckless, Cockburn, Boelio, & Perlman, 2010; Koch & Schmid‐Hempel, 2011), including humans (Kamada, Seo, Chen, & Núñez, 2013; Ley et al., 2006; Maynard, Elson, Hatton, & Weaver, 2012) wherein microbes can supplement host immune systems (Abt & Artis, 2013; Hooper, Littman, & Macpherson, 2012; McFall‐Ngai et al., 2013).

The net benefits of defensive mutualism are dependent upon the presence of pathogens (Clay, Holah, & Rudgers, 2005; King & Bonsall, 2017; Lively, Clay, Wade, & Fuqua, 2005). While hosts can benefit from microbe‐mediated protection, defensive symbionts can be less beneficial to the host in the absence of enemies, due to metabolic and physiological costs (King, 2019). For example, in the interaction of aphids and the bacterium *Hamiltonella defensa*, the host tissue is harmed by defensive toxins that protect against infection from parasitoids (Vorburger & Gouskov, 2011). In some cases, possessing protective microbes might be more beneficial to the host than investing in its own immune system (Martinez et al., 2016). From the perspective of the symbiont, it is most useful to its host under high pathogen prevalence and thus can persist in the host population (Palmer et al., 2008). Nevertheless, a stable symbiotic interaction is hypothesized to be evolved and maintained (Kwiatkowski & Vorburger, 2012) only when the host benefit of carrying defensive symbionts outweighs any costs. The interactions of obligate and defensive symbionts and hosts can be stable for millions of years (Moran, Tran, & Gerardo, 2005).

Not all environments are constantly pathogen‐rich, which might shift the balance of costs and benefits during defensive mutualisms, particularly during coevolutionary interactions (King & Bonsall, 2017). Pathogen prevalence can be spatially (King, Delph, Jokela, & Lively, 2009) or temporally variable, the latter in the case of seasonal epidemics (e.g., flu peaks each winter in the Northern Hemisphere (Finkelman, 2007) or rabies in North American skunks, which peaks in Autumn (Gremillion‐Smith & Woolf, 1988). Different environmental factors can influence disease transmission such as an increase in malaria risk in warmer regions after rainfall (Altizer, Dobson, Hosseini, Hudson, & Pascual, 2006) or an increase in contact rate and thus higher flu infection rate during the winter months (London & Yorke, 1973). The impact of other temporally heterogeneous factors on the strength and direction of selection on species interactions have been explored (oxygen concentration [Dey, Proulx, & Teotónio, 2016], resource availability [Friman & Laakso, 2011; Friman, Laakso, Koivu‐Orava, & Hiltunen, 2011; Hiltunen, Friman, Kaitala, Mappes, & Laakso, 2012], environmental productivity [Harrison, Laine, Hietala, & Brockhurst, 2013]). Whether the varied presence of pathogens can similarly alter selection for symbiotic interactions has been explored theoretically (Fenton, Johnson, Brownlie, & Hurst, 2011), but remains to be empirically tested.

Here, we examined the impact of temporal variation in pathogen infection on the evolution of microbe‐mediated protection. We used *Caenorhabditis elegans* as a worm host and allowed it to be colonized by a bacterium (*Enterococcus faecalis*) that protects against infection by *Staphylococcus aureus* (King et al., 2016). *Enterococcus faecalis* has been shown to be protective across animal microbiomes (Kommineni et al., 2015; Martín‐Vivaldi et al., 2010). It has been previously shown that *E. faecalis* can evolve to provide enhanced protection when residing in *C. elegans* hosts during constant pathogen infection (King et al., 2016; Rafaluk‐Mohr, Ashby, Dahan, & King, 2018). From this, we predict that variation in pathogen infection might limit the evolution of microbe‐mediated protection. In the present study, we experimentally copassaged *C. elegans* with protective *E. faecalis* and infected the host with evolutionary static pathogenic *S. aureus* at different intervals of host evolution. We also examined whether pathogen presence at the initial formation of the coevolving interaction is crucial to the evolution of protection. We show that enhanced microbe‐mediated protection emerged out of novel coevolutionary host–microbe interactions and during pathogen infection, regardless of its temporal variability or the time point of first infection. Enhanced protection was only effective during pathogen infection. If hosts survived infection, they could recover and had the same longevity and reproductive output across treatments. These results thus suggest that even occasional pathogen infection can select for defensive mutualism, revealing the potential for this phenomenon to be widespread in nature.

## 2 MATERIALS AND METHODS

### 2.1 Worm host and bacteria system

As a bacteriovore, *Caenorhabditis elegans* interacts constantly with a variety of bacteria either by feeding or by hosting them (Cabreiro & Gems, 2013; Garsin et al., 2001; Schulenburg & Ewbank, 2004). Consequently, *C. elegans* is an established model for studying innate immunity (Gravato‐Nobre & Hodgkin, 2005), as it can be infected with its natural (Jansson, 1994; Schulenburg & Ewbank, 2004) as well as opportunistic pathogens (Garsin et al., 2001; Tan, Mahajan‐Miklos, & Ausubel, 1999). Most pathogens are taken up orally by the worm (Marsh & May, 2012), and some can proliferate and colonize the worm gut (King et al., 2016; Rafaluk‐Mohr et al., 2018).

Naturally, *C. elegans* is a self‐fertilizing hermaphrodite (Brenner, 1974), but in this experiment obligate outcrossing worm populations (line EEVD00) with males and females (hermaphrodites that carry the *fog‐2(q71)* mutation) were used (Theologidis, Chelo, Goy, & Teotónio, 2014). This lineage was generated by Henrique Teotonio (ENS Paris) and encompasses the genetic diversity of 16 natural worm isolates (Theologidis et al., 2014). Worms were kept on Nematode Growth Medium (NGM), inoculated with *Salmonella*, hereafter referred to as food. Worms were infected with the pathogenic *S. aureus* (MSSA476; Holden et al., 2004), which is virulent and kills worm hosts by lysing the intestinal cells lining the gut wall (Sifri, Begun, Ausubel, & Calderwood, 2003). Worms were exposed to *E. faecalis* (OG1RF; Garsin et al., 2001), which was isolated from the human digestive system, but has been previously shown to colonize and proliferate in the host gut (Ford, Williams, Paterson, & King, 2017; King et al., 2016; Rafaluk‐Mohr et al., 2018), where it provides protection.

### 2.2 Experimental evolution—Design

Six single clones of *E. faecalis* (one for each of the six replicate populations) and a single population of *C. elegans* were the ancestors (hereafter referred to as the Ancestor) for all evolving populations. To account for potential differences in virulence, a stock of four clones of *S. aureus* was used for pathogen infections. Both *C. elegans* and colonizing *E. faecalis* were allowed to evolve in the presence of each other, while *S. aureus* was kept evolutionarily static. Infection with *S. aureus* was varied over host evolutionary time (indicated by purple in Table 1) to represent temporal heterogeneity in pathogen infection, including a range from always to every 2nd generation, every 5th generation, and never (Table 1). Moreover, we included differences in whether pathogens were present at the initial formation of the symbiotic interaction or later (2.1. vs. 2.2., and 5.1. vs. 5.2. in Table 1). Controls for laboratory adaptation were maintained for the host (No Protective Microbe control, NPM in Table 1) and *E. faecalis* (No Host Control, NHC in Table 1).

**TABLE 1:**
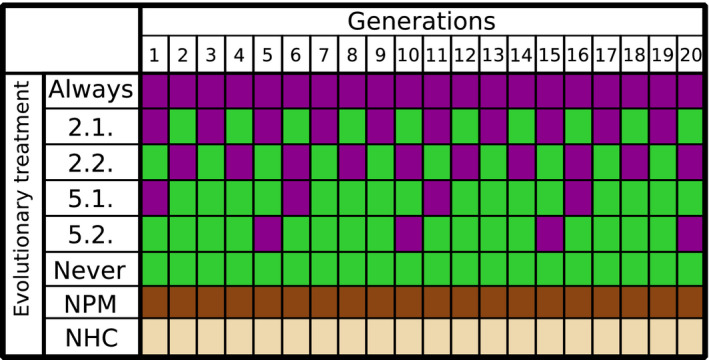
Experimental procedure for the evolution experiment

Columns indicate the number of experimental host generations (1–20), while rows show the eight treatments. Host generations were infected with *Staphylococcus aureus* (purple) or given food (green), while constantly coevolving with *Enterococcus faecalis*. Two controls for laboratory effects on host evolution (dark brown, No Protective Microbe, NPM) and *E. faecalis* evolution (light brown, No Host Control, NHC) were also included, where the NPM treatment was only ever exposed to food alone. Each evolutionary treatment consisted of six independent evolutionary replicates.

### 2.3 Experimental evolution—Culturing and passaging methods

At the start of each generation, worms were bleached as described previously and left in M9 buffer overnight for larvae to hatch (Stiernagle, 2006). Simultaneously, *E. faecalis* clones were cultured overnight in Todd‐Hewitt Broth (THB) in 600 µl at 30ºC, while food was cultured overnight in LB broth. Subsequently, 9‐cm NGM plates were inoculated with 300 µl of each overnight culture. Plates with freshly inoculated bacteria were dried at room temperature before approximately 1,000 L1 worms were added to each NGM plate. After these plates dried at room temperature, they were transferred to a 20ºC incubator and left for 48 hr. Simultaneously, a liquid culture of *S. aureus* was grown in THB from frozen stock, while a liquid culture of food was grown in LB, and both were incubated under shaking conditions at 30ºC. The following day, 100 µl of each overnight culture was spread on 9‐cm plates, *S. aureus* on Tryptone Soy Broth agar (TSB) plates and food on NGM plates, and incubated at 30ºC overnight. To transfer worms to the pathogen or food plates, nematodes were washed off the *E. faecalis* plates with M9 buffer and washed three times over small‐pore filters to remove all externally attached bacteria, as previously described (Jansen et al., 2015; Papkou et al., 2019; Rafaluk‐Mohr et al., 2018). Worms were infected with either *S. aureus* or exposed to food (Table 1) and left at 25ºC for 24 hr. After this time, worms were then washed off the plates with M9 buffer once more to plate them on NGM plates seeded with food for laying eggs. Roughly, 10% of these worms was crushed and plated on *E. faecalis* selective medium (TSB + 100 mg/ml rifampicin). The remaining worms were left on food plates for 48 hr to allow for egg laying.

To passage *E. faecalis*, roughly 100 *E. faecalis* colonies were picked and grown up shaking overnight in 600 µl THB at 30ºC, while worms were bleached and left to hatch overnight. This cycle was repeated for 20 experimental host generations.

All passaged worms and *E. faecalis* samples were cryopreserved at −80ºC. A proportion of the offspring of surviving worms were frozen in 40% DMSO, and 100 µl of *E. faecalis* liquid culture was mixed with 100 µl of glycerol before cryopreservation.

### 2.4 Host survival and fecundity assays

All assays were conducted at the end of the evolution experiment on archived samples. Plates were randomized and fully encoded during each experiment to ensure the experimenter was blind to different treatments while collecting data.

Basic procedures were adopted from the experimental evolution, but with the following alterations to keep the assays feasible with higher accuracy when scoring dead and alive worms: 400 L1 worms were exposed to 200 µl of food and *E. faecalis* on 6‐cm NGM plates, while 60µl of *S. aureus* overnight culture was used to inoculate 6‐cm TSB plates.

To assess microbe‐mediated protection of different combinations of worms and *E. faecalis*, 400 L1s were exposed to 50:50 mixtures of *E. faecalis* and food for 48 hr. Worms were then washed off these plates as described above and infected with *S. aureus* for 24 hr at 25°C. Survival in form of counting dead and alive worms was then scored.

To assess any long‐term fitness consequences after protective microbe exposure and pathogen infection, long‐term survival and fecundity were measured. Worms were exposed as described for the survival assays. Subsequently, five females and five males were picked onto 3‐cm food seeded NGM plates at 25°C and then transferred to new plates every 36 hr to avoid any confusion between offspring produced and original adults. At each time point, survival was scored. To measure fecundity, the number of worm eggs on the plates at 120 hr since bleaching was counted.

### 2.5 Statistical analysis

Statistical analyses were carried out with RStudio (version 1.1.463 for Mac), and graphs were created with the ggplot2 package (version 2.1.0) and edited with Inkscape (version 0.91). All host survival and fecundity data were analyzed with nested binomial mixed‐effects models (R package lme4), followed by a Tukey multiple comparison tests (R package multcomp). Life span data were analyzed with Kaplan–Meier log‐rank test with FDR correction for multiple testing.

## 3 RESULTS

**FIGURE 1:**
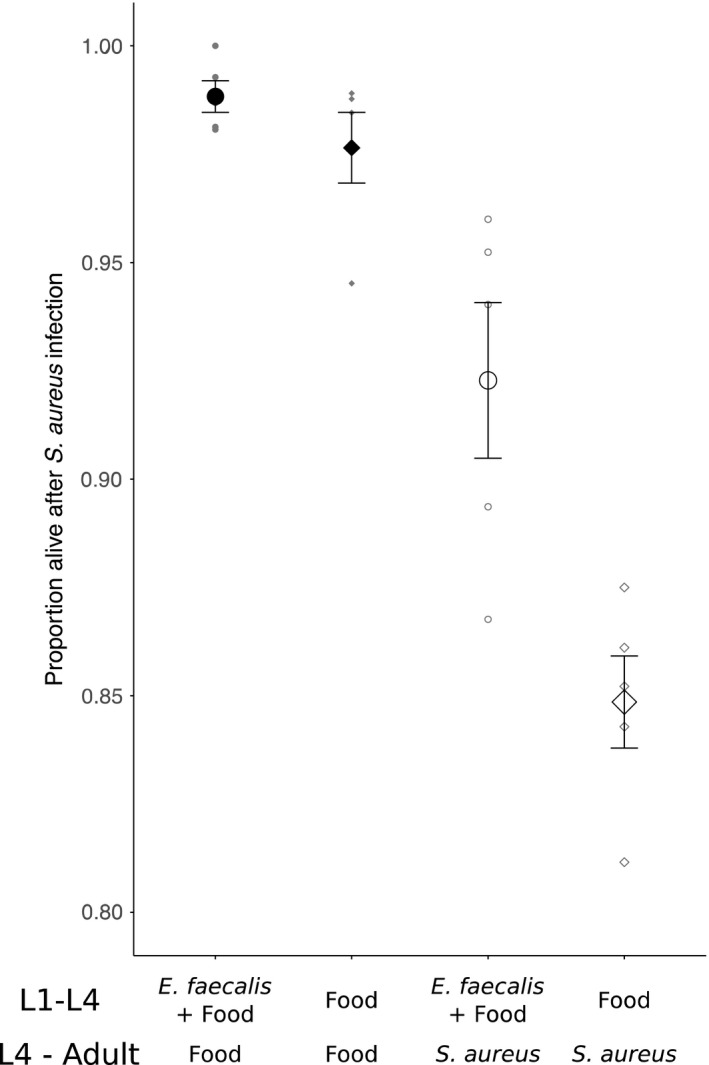
Host survival showing protective effects of *Enterococcus faecalis*. Early exposure of worms to *E. faecalis* (both ancestors) provides some degree of protection from the infection of *Staphylococcus aureus*. 24‐hr host survival levels reveal a benefit to *E. faecalis* colonization independent of pathogen presence or absence. Circles indicate those treatment being exposed to *E. faecalis* and food in the earlier stage (L1–L4), while squares indicate food alone treatment in the earlier stage (L1–L4). Filled symbols indicate those treatments being exposed to food in the later stage, while open symbols indicate those treatments being exposed to the pathogen *S. aureus* in the later stage. Each symbol indicates the mean ± *SE* of five replicates. Axis scales were chosen to be the same across all plots

Before the start of the evolution experiment, the starting conditions were tested. Confirming previous results, *E. faecalis* showed some spontaneous host‐protective potential against *S. aureus*. Worms raised on *E. faecalis* and food survived better than those raised on food alone, independent of food or pathogen present at the later stage (general linear model, *X* ^2^ = 10.205, *df* = 1, *p* = .001; Figure 1). Worms infected with *S. aureus* in later life survived worse than those being exposed to food (general linear model, *X* ^2^ = 119.643, *df* = 1, *p* < .001; Figure 1). These results demonstrate the beneficial and protective effects for the host after exposure to the protective microbe *E. faecalis*.

**FIGURE 2:**
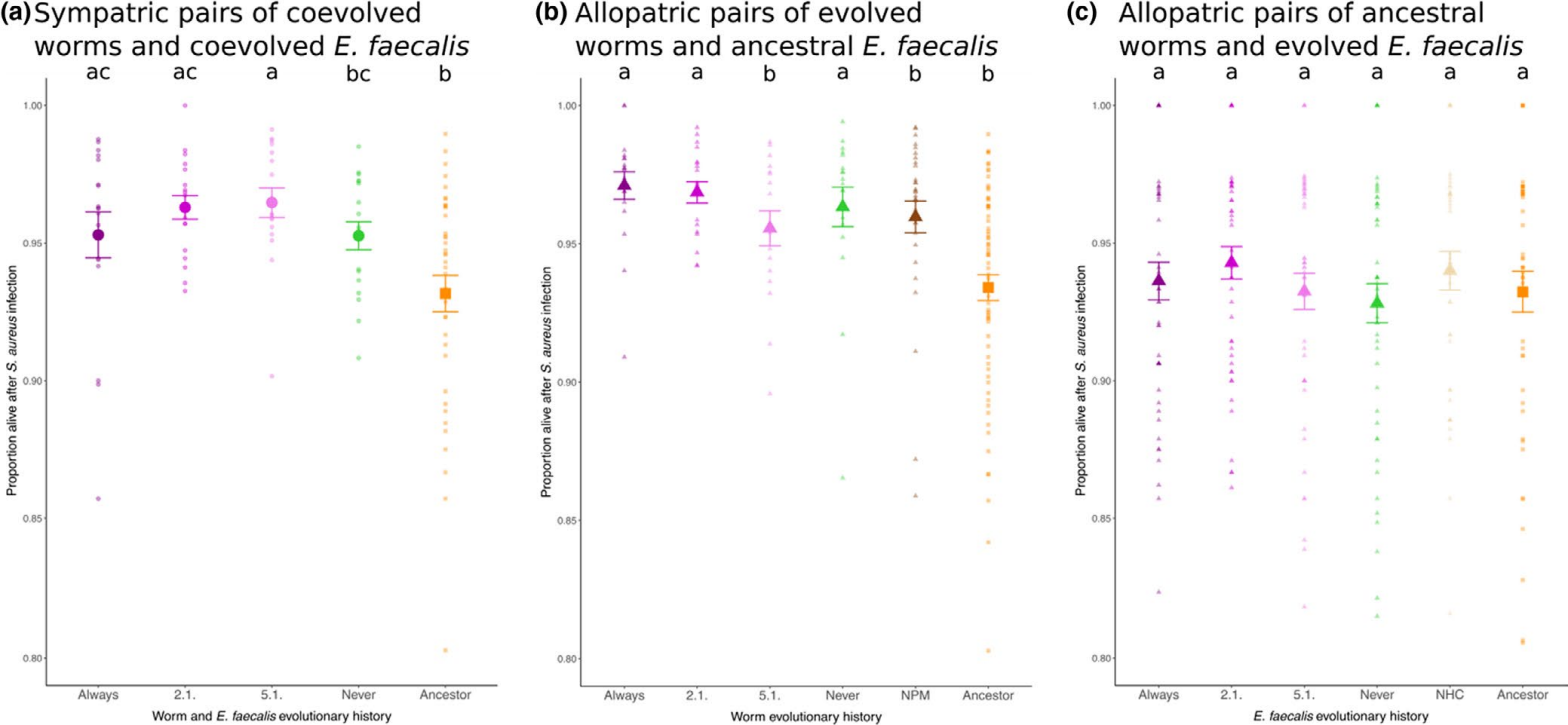
Host survival for coevolving sympatric and allopatric pairs of worms and *Enterococcus faecalis*. Microbe‐mediated protection was assessed for (a) sympatric pairs of coevolved worms and *E. faecalis*, (b) allopatric pairs of evolved worms and ancestral *E. faecalis*, and (c) allopatric pairs of ancestral worms and evolved *E. faecalis*. Bigger symbols represent mean ± *SE* and consist of six biological replicates and four technical replicates. Smaller symbols indicate the data distribution. Circles indicate sympatric pairs of coevolved *E. faecalis* and worms, squares indicate ancestral pairs of *E. faecalis* and worms, and triangles indicate allopatric pairs of *E. faecalis* and worms. Letters indicate results of a GLMM, followed by a Tukey post hoc test. The same letter indicates no significant difference. Axis scales were chosen to be the same across all plots

Infection with *S. aureus* over evolutionary time in the experiment led to the substantial enhancement of microbe‐mediated protection, with the evolutionary background of the sympatric pair of host and *E. faecalis* having a significant impact on host survival (mixed‐effects model, *X* ^2^ = 42.479, *df* = 4, *p* < .001; Figure 2a). Higher microbe‐mediated protection in comparison with the Ancestor occurred in all evolutionary histories involving pathogen presence across the temporal heterogeneity treatments in our evolution experiment (always, 2.1. and 5.1.). However, this did not occur in the pathogen absence (never) treatment. Host evolutionary history alone had a significant effect on host survival (mixed‐effects model, *X* ^2^ = 35.779, *df* = 5, *p* < .001; Figure 2b), but did not reveal the same pattern as for sympatric pairs. No effect of bacteria evolutionary history alone on infected host survival was observed (mixed‐effects model, *X* ^2^ = 3.2511, *df* = 5, *p* = .6613; Figure 2c). Taken together, enhanced microbe‐mediated protection evolved only as a product of coevolution and pathogen presence for sympatric pairs; this occurred regardless of the temporal heterogeneity.

**FIGURE 3:**
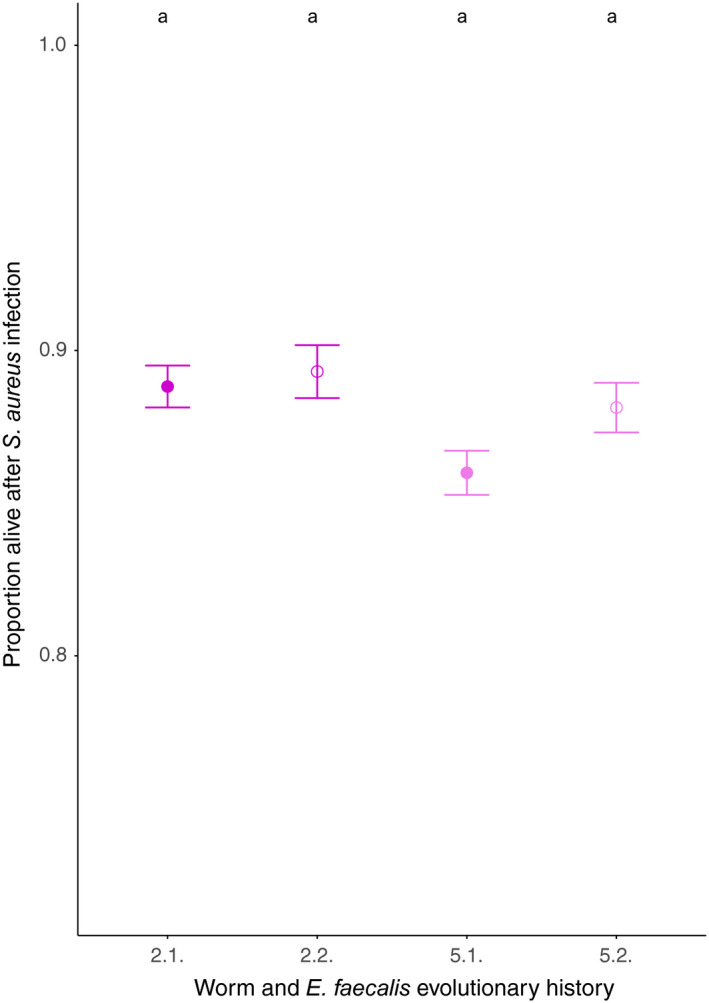
Host survival in evolutionary treatments differing in initial pathogen exposure time points. The time point of initial infection varied for infection to the pathogen every two generations (2.1. and 2.2) or every five generations (5.1. or 5.2.) but does not influence the outcome. Closed symbols indicate initial pathogen presence (host generation 1); open symbols indicate later pathogen presence (generation 2 for 2.1. and 2.2. and generation 5 for 5.1. and 5.2.). Bigger symbols represent mean ± *SE* and consist of six biological replicates and four technical replicates of the sympatric pairs. Smaller symbols indicate the data distribution. Letters indicate results of a GLMM, followed by a Tukey post hoc test. The same letter indicates no significant difference. Axis scales were chosen to be the same across all plots

As an additional form of pathogen heterogeneity, the impact of the timing of initial pathogen infection on the evolution of microbe‐mediated protection was investigated. An effect of different initial pathogen infection time points on host survival following pathogen infection was observed (mixed‐effects model: *X* ^2^ = 7.945, *df* = 3, *p* = .04716 Figure 3), although a Tukey post hoc test revealed no significant differences (Table S1).

**FIGURE 4:**
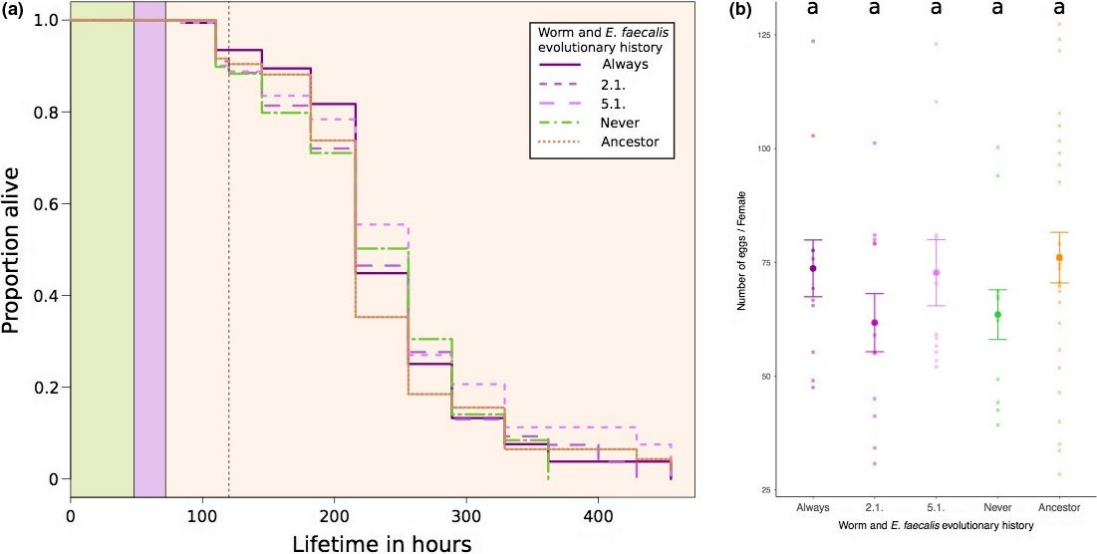
Long‐term survival and fecundity of *Enterococcus faecalis‐*colonized hosts that survived pathogen infection. (a) Long‐term host survival was measured. Survival curves for sympatric pairs of worms and *E. faecalis* are shown as Kaplan–Meier estimates. Worms were exposed to *E. faecalis* and food (green), and then to *Staphylococcus aureus* (purple), and long‐term survival was monitored on food (orange). The dotted line indicates the time point at which fecundity was measured. (b) Number of eggs/female across sympatric pairs of coevolved worms and *E. faecalis*. Bigger symbols represent mean ± *SE* and consist of six biological replicates and four technical replicates. Smaller symbols indicate the data distribution. Circles indicate sympatric pairs of coevolved *E. faecalis* and worms; squares indicate ancestral pairs of *E. faecalis* and worms. Letters indicate results of a GLMM, followed by a Tukey post hoc test. The same letter indicates no significant difference

Furthermore, we investigated the long‐term consequences to hosts colonized by *E. faecalis* after 24 hr of pathogen infection. No significant differences were observed in the long‐term survival postinfection of worm hosts colonized by their sympatric *E. faecalis* across treatments (Kaplan–Meier log‐rank test, FDR‐corrected, all comparisons *p* > .05, Figure 4a). In addition, we did not find significant differences in fecundity among sympatric host– *E. faecalis* pairs (mixed‐effects model, *X* ^2^ = 3.9418, *df* = 4, *p* = .4278, Figure 4b).

## 4 DISCUSSION

It has been shown that hosts receive the greatest benefits from protective microbes under constant pathogen infection. We hypothesized that variation in pathogen presence over time would limit the evolution of microbe‐mediated protection due to the reduced benefits to the host and bacterial symbiont. In our study, enhanced pathogen defense emerged out of host–symbiont coevolutionary interactions only when pathogens were present, independent of the interval or initial presence of the pathogen. Notably, the ultimate strength of microbe‐mediated protection that evolved was not impacted by the number of host generations between pathogen infections, the proportion of generations infected, or the presence of the pathogen at the first host–microbe interaction. These results suggest that resident microbes can be a form of transgenerational immunity against rare pathogen infections.

We found that microbe‐mediated protection is maintained even in the prolonged absence of pathogen, but that pathogen presence is necessary for microbe‐mediated protection to evolve, as previously hypothesized (Clay et al., 2005; King & Bonsall, 2017; Lively et al., 2005). This result is unlike previous work showing that the scale of heterogeneity in abiotic conditions can affect the strength of selection for traits in some symbiotic interactions (Harrison et al., 2013). This discrepancy is potentially due to costs in our symbiotic system being ameliorated (at least in terms of host survival) in well‐provisioned hosts, as hosts are provided with food alongside *E. faecalis* and are thus rescued from starvation (also see Dasgupta et al., 2019). Although protective symbionts can incur costs (e.g., Vorburger & Gouskov, 2011) for their hosts, with potential for impacts on coevolutionary interactions (King & Bonsall, 2017), it is possible that potential costs of bacterial colonization might be only detectable when hosts are stressed (Lively, 2006) or that the costs were not strong enough for us to detect (Little, Carius, Sakwinska, & Ebert, 2002). Different measures of cost remain to be explored (e.g., life span in the complete absence of a protective microbe and a pathogen). Higher protection also does not always come with higher costs, as found in the black bean aphid–*Hamiltonella defensa* interaction (Cayetano, Rothacher, Simon, & Vorburger, 2015). Thus, protective traits in an organism's commensal microbiota could be selected for under pathogen infection and easily maintained in subsequent uninfected generations.

Microbe‐mediated protection was strongest between sympatric pairs when pathogens were present over evolutionary time, consistent with previous findings (Rafaluk‐Mohr et al., 2018). In our study, protection emerged during coevolution after only 20 host generations, and not due to the independent evolution of either interacting species, but due to the coevolution of both species (King & Bonsall, 2017). The time scale of these interactions is short compared to the longer shared evolutionary histories shared by other defensive mutualisms (Jousselin, Rasplus, & Kjellberg, 2003; Quek, Davies, Itino, & Pierce, 2004; Shoemaker et al., 2002). Nevertheless, our findings reveal the potential for microbe‐mediated protection to become enhanced during the formation of a coevolving host–microbiota relationship.

In conclusion, our results show that enhanced protection in host–microbe interactions can rapidly evolve and be maintained even under infrequent pathogen infection, suggesting that resident microbes can be a form of stable, transgenerational immunity. The protective benefit of an organism's microbiota might remain undetected for several host generations until pathogens re‐emerge. Future research on the failure of pathogens transmit within host populations should consider the contribution of the protective microbiota to prevent disease spread.

## CONFLICT OF INTEREST

The authors declare no conflict of interest.

## AUTHOR CONTRIBUTION

**Anke Kloock:** Conceptualization (equal); Data curation (equal); Formal analysis (lead); Investigation (equal); Visualization (lead); Writing‐original draft (lead). **Michael B. Bonsall:** Conceptualization (supporting); Data curation (supporting); Formal analysis (supporting); Supervision (equal); Writing‐review & editing (equal). **Kayla C. King:** Conceptualization (equal); Data curation (supporting); Formal analysis (supporting); Funding acquisition (lead); Supervision (equal); Writing‐review & editing (lead).

## Supporting information

Appendix S1

## Data Availability

All evolved worm and bacteria strains are cryopreserved and can be provided upon request. Raw data and all scripts that were used for statistical analysis are available via the following link: https://osf.io/vpm9b/.
