# Supplementary material for: Evolution and maintenance of microbe‐mediated protection under occasional pathogen infection
Source: Ecol Evol. 2020 Jul 16;10(16):8634–42. doi: 10.1002/ece3.6555 (PMC7452762; doi:10.1002/ece3.6555)
Supplement: Supplementary file 1 — Appendix S1 [file ECE3-10-8634-s001.docx]

# Appendix

## Experimental Procedure


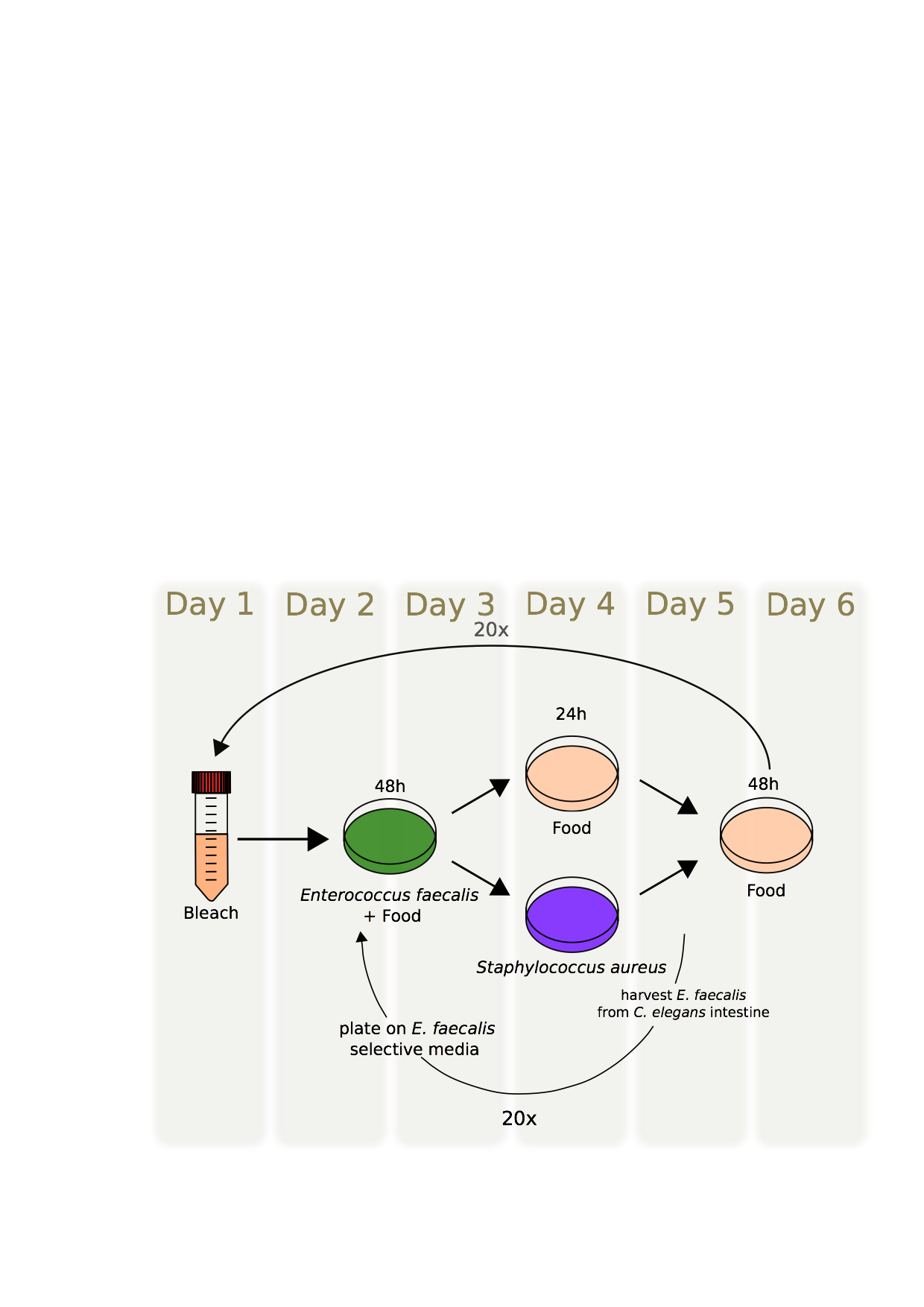


Figure A1: The experimental procedure of the evolution experiment in detail.

The procedure of the evolution experiment can be seen in Figure S1 and will be the following:

1. On the first day of each generation, worms were bleached(Stiernagle, 2006). During this step worms were removed from the plates by washing with M9 buffer. Afterwards a 50:50 mixture of 10% NaClO and 5M NaOH was used to bleach all the remaining bacteria in the solution and to release eggs from bleached adult worms. Only eggs survive this step and were then left in the M9 buffer over-night. All bleached eggs hatched overnight, but do not develop any further and thus lead to increased synchronisation for each worm population. Every bleach solution was plated out on a TSA plate to control for carried over contaminations. 100 colonies of *E. faecalis* were picked from the plates that were grown on *E. faecalis* selective medium for 48 hours at 30°C. These colonies were picked into 600μl of THB and then grown up over-night. Simultaneously, a single colony of *Salmonella* food was picked and added to 25ml of LB broth to be grown under shaking conditions over night.
2. On the second day of each generation, the overnight shaken and hatched worms were exposed to 600μl of a 50:50 mixture of *E. faecalis* and food. At this step the population size was adjusted to only contain 1000 individuals. Worms remained on these plates with *E. faecalis* for 48 hours.
3. On the third day of each generation, TSA plates to which worms were going to be exposed on day 4 were inoculated with 100μl of either *S. aureus* or food and were incubated at 30°C overnight.
4. On the fourth day of each generation, worms were washed off the plates seeded with
   *E. faecalis* by filter tip washing. For this purpose, worms were washed off the plates with twice 1.5ml of M9 buffer +1% Triton X100, as previously described(Jansen et al., 2015; Papkou et al., 2019; Rafaluk-Mohr et al., 2018).This worm and bacteria suspension was spun down for 1 minute, 1 ml of the supernatant was discarded and the rest of the pellet was pipetted on the top of a filter of a filter tip and spun down for 3 min. Worms were left on the top of the filter were washed with 400μl of M9 three times, before being re-suspended in 100μl of M9 to bring onto plate. During this method, most of the externally attached bacteria are washed off the worms to ensure that worm survival can be attributed to gut colonization of *E. faecalis* and not external attachment of the protective microbe. *Enterococcus faecalis* remains in the worm’s intestine and will establish a protective effect. After worms were transferred to the plates containing either *S. aureus* or food, all plates were moved to 25°C.
5. On the fifth day of each generation, worms were washed off the plates again by filter tip washing (as described for day 4 and previously (Jansen et al., 2015; Papkou et al., 2019; Rafaluk-Mohr et al., 2018)). Worms were left on plates seeded with food at 20°C for 48 hours to lay eggs. The amount of transferred bacteria (either *S. aureus* or food can be neglected to have any influence on the further development of the worms. These plates were then used for bleaching on day 1 of the following generation. 10% of the worm mixture was separated and used to isolate *E. faecalis*. For this purpose, the suspension of worms was crushed and then plated on TSA plates with Rifampicin, as the *E. faecalis* strain carries a Rifampicin resistance. The plated gut content was allowed to grow at 30°C for 48 hours.

## Statistical results:

Table A1: All statistical results summarized, including the statistical test, the specifics associated with each test, the relevant degrees of freedom and p-values.

| Figure | Results |
| --- | --- |
| 1 | Binomial GLM   \| Comparison \| X^2^ \| Degrees of Freedom \| p-value \| \| --- \| --- \| --- \| --- \| \| L1-L4 stage \| 10.205 \| 1 \| 0.001401 \| \| L4 – Adult \| 119.643 \| 1 \| <2.2 x10^-16^ \| \| Interaction \| 0.053 \| 1 \| 0.871 \| |
| 2A | Binomial GLM  Evolution effect: X^2^: 42.479, Df=4, p=1.327x10^-8^  Tukey Post-hoc comparisons:   \| Comparison \| Estimate Std. \| Error \| z-value \| p-value \| \| --- \| --- \| --- \| --- \| --- \| \| Always – Ancestor \| -0.5490 \| 0.1354 \| -4.055 \| <0.001 \| \| 2.1. - Ancestor \| -0.5902 \| 0.1348 \| -4.378 \| <0.001 \| \| 5.1. – Ancestor \| -0.8224 \| 0.1448 \| -5.680 \| <0.001 \| \| Never – Ancestor \| -0.3174 \| 0.1290 \| -2.460 \| 0.0985 \| |
| 2B | Binomial GLM  Evolution effect: X^2^: 35.779, Df=5, p=1.051x10^-6^  Tukey Post-hoc comparisons:   \| Comparison \| Estimate Std. \| Error \| z-value \| p-value \| \| --- \| --- \| --- \| --- \| --- \| \| Always – Ancestor \| -0.92335 \| 0.19399 \| -4.760 \| <0.001 \| \| 2.1. - Ancestor \| -0.84208 \| 0.18860 \| -4.465 \| <0.001 \| \| 5.1. – Ancestor \| -0.49494 \| 0.18527 \| -2.671 \| 0.0791 \| \| Never – Ancestor \| -0.78893 \| 0.19395 \| -4.068 \| <0.001 \| \| NPM – Ancestor \| -0.52794 \| 0.18847 \| -2.801 \| 0.0560 \| |
| 2C | Binomial GLM  Evolution effect: X^2^: 3.2511, Df=5, p= 0.6613 |
| 3 | Binomial GLM  Evolution effect: X^2^: 7.945, Df=3, p= 0.04716   \| Comparison \| Estimate Std. \| Error \| z value \| p-value \| \| --- \| --- \| --- \| --- \| --- \| \| 2.2. - 2.1. \| 0.05039 \| 0.10064 \| 0.501 \| 0.9576 \| \| 5.1. - 2.1. \| -0.26298 \| 0.12387 \| -2.123 \| 0.1414 \| \| 5.2. - 2.1. \| -0.07281 \| 0.12634 \| -0.576 \| 0.9373 \| \| 5.1. - 2.2. \| -0.31337 \| 0.12399 \| -2.527 \| 0.0538 \| \| 5.2. - 2.2. \| -0.12320 \| 0.12646 \| -0.974 \| 0.7585 \| \| 5.2. - 5.1. \| 0.19017 \| 0.09467 \| 2.009 \| 0.1795 \| |
| 4A | Kaplan Meier Estimates   \| Comparison \| p-value \| FDR corrected p-value \| \| --- \| --- \| --- \| \| Always – Ancestor \| 0.16086355 \| 0.3217271 \| \| 2.1. - Ancestor \| 0.71526638 \| 0.7152664 \| \| 5.1. – Ancestor \| 0.04131051 \| 0.1652421 \| \| Never – Ancestor \| 0.04131051 \| 0.7152664 \| |
| 4B | Binomial GLM  Evolution effect: X^2^: 3.8418, Df=4, p= 0.4278 |
